# 

**Published:** 1906-02

**Authors:** 


### ATLANTA JOURNAL-RECORD OF MEDICINE.

Successor to Atlanta Medical and Surgical Journal, Established 1855, and Southern Medical Record, Established 1870.

Vol. VII. Atlanta, Ga., February, 1906. No. 11.

